# Clinical epidemiology of community-acquired pneumonia in children before, during, and after the COVID-19 pandemic, and independent risk factors analysis for severe community-acquired pneumonia

**DOI:** 10.7189/jogh.15.04212

**Published:** 2025-09-19

**Authors:** Xiaoou Li, Lingzhi Li, Xinjiang An, Jing Tian, Bianba Zhuoga, Lianhua Jin, Huijing Jin, Xufang Li, Ying Liu, Jiamin Li, Wanzhen Mei, Ping Liu, Jinyong Pan, Zhaotang Lin, Yusheng Pang, Xiao Wu, Qian Peng, Xiaoping Hu, Xuewen Su, Xiaoning Wang, Lin Feng, Haitao Zhang, Dehua Zhang, Shutong Yang, Zhenpeng Lu, Wenqian Chen, Bing He

**Affiliations:** 1 Department of Pediatrics, Renmin Hospital of Wuhan University, Wuhan, China; 2 Department of Cardiology, Xu Zhou Children's Hospital, Xuzhou, China; 3 Department of Pediatrics, People's Hospital of Tibet Autonomous Region, Lasa, China; 4 Department of Pediatric Cardiovascular, Children's Medical Center, The First Hospital of Jilin University, Changchun, China; 5 Department of Cadre Ward, The First Hospital of Jilin University, Changchun, Jilin, China; 6 Department of Pediatrics, Changzhi City People’s Hospital, Changzhi, China; 7 Department of Pediatrics, Peking University Shenzhen Hospital, Shenzhen, China; 8 Department of Pediatric Cardiovasology, Children’s Medical Center, The Second Xiangya Hospital, Central South University, Changsha, China; 9 Department of Pediatrics, First Affiliated Hospital of Shihezi University School of Medicine, Shihezi, China; 10 Department of Pediatrics, The First Affiliated Hospital of Guangxi Medical University, Nanning, China; 11 Department of Pediatrics, Sichuan Academy of Medical Sciences & Sichuan Provincial People’s Hospital, Chengdu, China; 12 Department of Pediatrics, Inner Mongolia People's Hospital, Hohhot, China; 13 Department of Pediatrics, The Second Hospital of Hebei Medical University, Shijiazhuang, China; 14 Pediatric Internal Medicine Department, The Second Affiliated Hospital of Harbin Medical University, Harbin, China

## Abstract

**Background:**

The COVID-19 pandemic may have created an ‘immunity debt’, altering the epidemiology and aetiology of paediatric community-acquired pneumonia (CAP). However, multi-centre data on these changes and contemporary risk factors for severe CAP remain limited. We therefore aimed to analyse clinical characteristics and pathogen composition of CAP in children before, during, and after the COVID-19 pandemic, and to identify independent risk factors associated with severe CAP.

**Methods:**

We collected clinical data from paediatric inpatients diagnosed with CAP at 13 hospitals in 13 provinces of China between August and October 2018 2018 (before the COVID-19 pandemic), 2020 (during the pandemic), and 2023 (after the pandemic). We used a multivariate COX regression model to identify independent risk factors for severe CAP.

**Results:**

Our sample comprised 5180 children hospitalised with CAP, with fever and cough being the main clinical symptoms. The number of school-aged children with CAP was significantly higher after compared to before and during the pandemic, while cellular immune function was significantly reduced (*P* < 0.05). The proportion of viral infections increased from 7.4% before to 20.1% after the pandemic; mycoplasma infections rose significantly from 36.3% to 41.9%, while other bacterial infections increased from 4.9% to 9.7%. The proportion of bacterial infection was highest among infants (11%) and toddlers (9.4%). Viral infection (18.9%), virus-viral co-infections (3.1%), viral-bacterial co-infections (3.3%), and bacteria-bacterial co-infections (1.8%) were more common among preschool children, while mycoplasma infections (59.8%), mycoplasma-viral co-infections (10.5%), or mycoplasma-bacterial co-infections (4.6%) were more frequent among school-aged children. We identified wheezing (hazard ratio (HR) = 1.551), chest pain (HR = 3.144), haemoptysis (HR = 4.854), *Klebsiella pneumoniae* (HR = 4.236), *Acinetobacter baumannii* (HR = 4.432), influenza B virus (HR = 2.338), and adenovirus infection (HR = 2.895) as risk factors for severe CAP.

**Conclusions:**

We observed a shift in the epidemiology of CAP in children following the pandemic, with altered immune responses and pathogen profiles. These findings emphasise the need for targeted treatment strategies in paediatric patients in the post-COVID-19 context to reduce the burden of severe CAP.

The COVID-19 pandemic has fundamentally changed the dynamics of respiratory infections globally, leading to a need for closer monitoring of epidemiological trends. Since the outbreak, a series of measures, collectively referred to as non-pharmaceutical interventions (NPIs), have been implemented worldwide to mitigate the spread not only of COVID-19 [1], but have also reduced the transmission of other respiratory pathogens [2–4]. Decreased exposure to bacteria and viruses has substantially altered the development of natural immunity, creating a kind of ‛immunity debt’ [5]. With the gradual lifting of NPIs in China by late 2022, the incidence of respiratory diseases, including infections caused by *Mycoplasma pneumoniae* (MP), has increased significantly, with many cases showing severe clinical manifestations [6]. In the past, vaccination programmes have played a crucial role in shaping these trends. For instance, the widespread use of pneumococcal and influenza vaccines has been associated with a declining incidence of bacterial pneumonia and influenza-related complications in paediatric populations [7]. It is precisely because of the key role these programmes played that decreases in routine vaccination rates and coverage of national immunisation programmes during the COVID-19 pandemic inevitably led to increases in susceptibility to vaccine-preventable diseases among the general population [8].

Paediatric community-acquired pneumonia (CAP) was already recognised as a significant cause of morbidity and mortality among children under five years before the pandemic, with notable variations in incidence rates influenced by factors such as seasonality and pathogen diversity [9,10]. More than 120 million children under the age of five are affected by CAP worldwide each year, with over 10% suffering from severe CAP (SCAP) [9,10]. After the pandemic, or more precisely, from August to October 2023, there was a significant increase in the number of children diagnosed with CAP exceeding the levels in the same seasonal period before the pandemic [11]. As CAP is the most prevalent disease among hospitalised children, identifying its epidemiology and pathogens is vital. The distribution of CAP-associated pathogens can vary based on factors such as age, gender, geographic differences, and disease severity, with seasonal variations also observed in their composition.

One national surveillance study conducted in China between 2009 and 2020 investigated the infection and co-infection patterns of CAP in patients of various ages [12]. Yet due to gaps in the literature, it remains essential to investigate the pathogens associated with CAP in hospitalised children, including both bacterial and viral infections, as well as their co-infection patterns.

Therefore, we aimed to examine the prevalence and pathogenic characteristics of CAP stratified by time and age, and to assess the impact of COVID-19 on the pathogen composition and immune function in children with CAP.

## METHODS

### Study participants

We conducted a retrospective study on paediatric inpatients diagnosed with CAP across 13 hospitals in 13 provinces of China, spanning from north to south, including Hubei, Jiangsu, Tibet, Jilin, Shanxi, Shenzhen, Hunan, Xinjiang, Guangxi, Sichuan, Inner Mongolia, Hebei, and Heilongjiang. We selected these hospitals deliberately to ensure a representative sample of the paediatric population, capturing the demographic and epidemiological diversity of cases across the country. To facilitate direct seasonal comparison, the collected data spanned three identical periods: from August to October in 2018 (before the pandemic), 2020 (during the pandemic), and 2023 (after the pandemic).

We excluded outpatients; patients with bronchial asthma, pulmonary tuberculosis, or bronchopulmonary dysplasia; patients with severe diseases complications of other organs such as those affecting the heart, brain, liver, or kidneys, before admission; patients with other immunodeficiency diseases or tumours; patients with nosocomial pneumonia; and patients with incomplete data.

### Sample size

Hospitals were selected through stratified sampling to ensure they provided a representative sample of the geographic diversity across China. We calculated the required sample size based on the total population size (*i.e.* the total number of children in the country); the confidence level (typically set at 95%); the margin of error (±5%); and the expected prevalence rate. When calculating at the provincial level, we used the proportional allocation method expressed as ‘sample size = Z^2^ × p (1−p)/e^2^’ where ‛Z’ is the Z-value corresponding to the confidence level (1.96 for a 95% confidence level), ‛p’ is the expected prevalence rate (the prevalence rate for CAP is assumed to be 0.05), and ‛e’ is the allowable sampling error (*e.g.* 0.05 denotes ±5%). Using this formula, we calculated that 73 samples per province would be the minimum required.

### Clinical symptom classification and CAP diagnostic criteria

We classified clinical symptoms based on the standardised criteria for diagnosing CAP in children. Primary symptoms were recorded upon patient admission and were evaluated by attending paediatricians. Here, we focussed on fever, cough, dyspnoea, wheezing, chest pain, thoracodynia, fatigue, haemoptysis, and any gastrointestinal symptoms.

The CAP was diagnosed by paediatricians according to the World Health Organization guidelines [13]. All participating hospitals are major medical institutions known for their capacity to handle paediatric cases. To ensure consistency and reliability of diagnoses, we conducted multiple training sessions for the paediatricians from each participating centre who were responsible for case selection and data collection. These sessions standardised the application of the diagnostic and case selection criteria across all study sites. All patients received chest X-rays or chest CT scans to confirm the CAP diagnosis. For the former, pneumonia was indicated by the presence of consolidation (dense or fluffy opacity, with or without air bronchograms), infiltrates (linear or patchy alveolar or interstitial densities), or pleural effusion. For the latter, the CT score was derived from the degree of infection involvement in the five lung lobes, where the extent of involvement for a single lung lobe was categorised as 0% (0 points), 1–25% (1 point), 26–50% (2 points), 51–75% (3 points), and 76–100% (4 points), resulting in a total score of up to 20 points. As described previously [14], a chest CT score >1 point indicates pneumonia, while ≥8 points indicates SCAP. Moreover, the Chinese Guidelines for CAP management in children recommend classifying CAP as severe when multiple lobes are involved or if a single lobe shows involvement of ≥2/3 [15].

### Data collection

We obtained baseline data from children with CAP, including age, gender, medical history, clinical symptoms, complications, and chest imaging findings. We collected blood samples on the day of admission to evaluate cellular immune function (lymphocyte counts of CD3^+^, CD4^+^, CD8^+^, and CD19^+^) and humoral immune function (levels of IgG, IgA, IgM, Complement C3, and Complement C4). We took nasopharyngeal swabs within 24 hours of admission and analysed them using RT-PCR to detect the nucleic acids of influenza A virus, influenza B virus, parainfluenza virus, rhinovirus, adenovirus, respiratory syncytial virus (RSV), metapneumovirus, coronavirus, Boca virus, and MP. We also collected induced sputum samples within 24 hours of admission for bacterial culture and nucleic acid detection of eight bacteria: *Streptococcus pneumoniae*, *Staphylococcus aureus*, *Haemophilus influenzae*, *Klebsiella pneumoniae*, *Pseudomonas aeruginosa*, *Acinetobacter baumannii*, *Stenotrophomonas maltophilia*, and *Moraxella catarrhalis*. We analysed serum samples for Epstein-Barr virus (EBV) antibodies, where positive results for EBVCA-IgM/VCA-IgG or negative results for EA-IgG/NA-IgG indicated primary infection [16]. Furthermore, we tested serum samples for fungal infection using the fungal G test and galactomannan test.

### Statistical analysis

We reported categorical variables as numbers and percentages, and compared them with the Pearson’s χ^2^ test or Fisher’s exact test. We presented continuous data as medians with interquartile ranges and analysed them across groups using the Kruskal-Wallis test. We defined the occurrence time for the event of SCAP as the duration from onset until the event occurred. To identify independent risk factors, we performed multivariate Cox regression analysis. Initially, we established a univariate model (model 1) to identify potential variables by using Pearson’s χ^2^ test, Fisher’s exact test, or the Kruskal-Wallis test. Subsequently, variables with *P* < 0.1 from the univariate analysis, along with age and gender, were used to construct a multivariate Cox regression model (model 2). Finally, we established the final model (model 3) after adjusting for confounding factors (such as the complications of SCAP and hospitalisation duration) and incorporating clinically relevant factors with *P*-values around 0.1 (such as haemoptysis). We verified the proportional hazards assumption using Kaplan-Meier survival curves and conducted sensitivity analyses using the backward elimination method to further validate the contributions of individual variables in the model. We also verified the performance of the risk factor model using receiver operating characteristic curve analysis. A two-sided *P*-value <0.05 was considered statistically significant. We generated graphics using *R*, version 4.4.1 (R Core Team, Vienna, Austria), and conducted statistical analyses using SPSS, version 20.0 (SPSS Inc., Chicago, IL, USA).

## RESULTS

### Clinical epidemiological characteristics of children with CAP before, during, and after the pandemic

#### Analysis of baseline data

**Figure 1:**
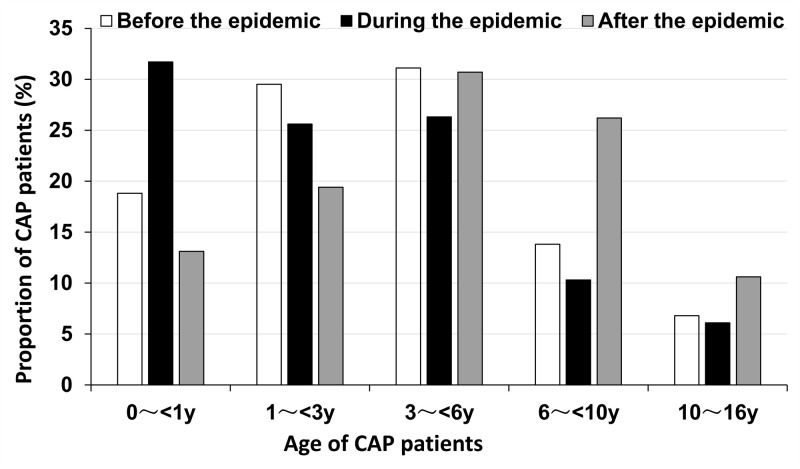
Age characteristics of children with CAP. CAP – community-acquired pneumonia.

We included 5180 children diagnosed with CAP, comprising 2878 males and 2302 females. They came from 13 provinces, with each contributing between 200 and 400 cases, thereby adhering to international sampling principles, which require 3000 to 5000 samples [17], ensuring representation of the national population. They were between 1 month and 16 years old, with a median age of 3.1 years (interquartile range = 1.0–6.1) (**Table 1**). There were 907 (17.5%) infants (<1 year old), 1201 (23.2%) toddlers (1–3 years old), 1560 (30.1%) preschoolers (3–6 years old), 1054 (20.3%) school-age children (6–10 years old), and 458 (8.9%) were adolescents (10–16 years old). The number and median age of children with CAP hospitalised after the pandemic were significantly higher than before and during the pandemic. Specifically, the number of children over six years of age with CAP after the pandemic was significantly greater than before and during the pandemic (*P* < 0.001), although the proportion of children under six years old affected by CAP remains substantial (**Figure 1**).

**Table 1:** Baseline characteristics of the study population*

Characteristics	Before the pandemic, n = 1459	During the pandemic, n = 785	After the pandemic, n = 2936	*P*-value†
Sex				
*Male*	810 (55.5)	478 (60.9)‡	1590 (54.2)‖	0.003
*Female*	649 (44.5)	307 (39.1)	1346 (45.8)	
Age in years, MD (IQR)	3.0 (1.0, 5.0)	1.9 (0.7, 4.0)‡	4.0 (2.0, 7.0)§	<0.001
*<1*	274 (18.8)	249 (31.7)‡	384 (13.1)§	<0.001
*1–3*	431 (29.5)	201 (25.6)	569 (19.4)§	<0.001
*3–6*	453 (31.1)	206 (26.3)	901 (30.7)‖	0.036
*6–10*	202 (13.8)	81 (10.3)‡	771 (26.2)§	<0.001
*10–16*	99 (6.8)	48 (6.1)	311 (10.6)§	<0.001

IQR – interquartile range, MD – median

*Presented as n (%) unless specified otherwise.

†Pearson’s χ^2^ test.

‡*P* < 0.05, compared with before the pandemic.

§*P* < 0.05, compared with before and during the pandemic.

‖*P* < 0.05, compared with during the pandemic.

#### Analysis of clinical symptoms and complications

Fever and cough were the primary clinical symptoms observed in children with CAP (**Table 2**). The incidence of fever after the pandemic was significantly greater than before and during the pandemic (*P* < 0.001), while the prevalence of cough was higher before and after the pandemic than in the pandemic period (*P* < 0.001). The overall prognosis for children with CAP in this study was favourable, with no deaths. The incidence of complications, such as respiratory failure and heart failure, was low, with the most frequently observed complication being liver function impairment. The rates of liver function impairment and heart failure in children with CAP after the pandemic were significantly lower than those observed before and during the pandemic (*P* < 0.001). The peak body temperature, median CT scores, and proportion of SCAP cases (CT score ≥8) after the pandemic were also significantly higher compared to those before and during the pandemic (*P* < 0.001). Lastly, the incidence of lung consolidation in children with CAP after the pandemic was significantly greater than that recorded during the pandemic (*P* < 0.001).

**Table 2:** Clinical symptoms and complications of the study population*

Characteristics	Before the pandemic, n = 1459	During the pandemic, n = 785	After the pandemic, n = 2936	*P*-value†
Symptom				
*Fever*	1032 (70.7)	434 (55.3)‡	2375 (80.9)§	<0.001
*Cough*	1433 (98.2)	737 (93.9)‡	2904 (98.9)‖	<0.001
Complication				
*Respiratory failure*	22 (1.5)	19 (2.4)	37 (1.3)	0.06
*Heart failure*	27 (1.9)	14 (1.8)	15 (0.5)§	<0.001
*Myocardial injury*	80 (5.5)	46 (5.9)	202 (6.9)	0.169
*Liver function impairment*	111 (7.6)	75 (9.6)	160 (5.4)§	<0.001
*Kidney function impairment*	9 (0.6)	8 (1.0)	18 (0.6)	0.443
*Coagulation dysfunction*	15 (1.0)	8 (1.0)	23 (0.8)	0.656
*Bronchiolitis obliterans*	3 (0.2)	3 (0.4)	3 (0.1)	0.166†
*Pneumothorax*	1 (0.07)	0	2 (0.07)	1.000†
Peak body temperature in °C, MD (IQR)	39.0 (38.5, 39.4)	38.9 (38.2, 39.3)	39.0 (38.5, 39.5)§	<0.001
Total fever duration, days	5.0 (3.0, 7.0)	3.0 (2.0, 5.0)‡	5.0 (3.0, 7.0)‖	<0.001
CT score	4 (2, 6)	4 (2, 5)	5 (3, 7)§	<0.001
CT score ≥8	68(8.9)	13 (3.6)‡	208 (13.0)§	<0.001
Lung consolidation	315 (21.6)	88 (11.2)‡	672 (22.9)‖	<0.001

IQR – interquartile range, MD – median

*Presented as n (%) unless specified otherwise.

†Pearson’s χ^2^ test with Fisher’s exact test. Other comparisons were conducted using the Pearson’s χ^2^ test.

‡*P* < 0.05, compared with before the pandemic.

§*P* < 0.05, compared with before and during the pandemic.

‖*P* < 0.05, compared with during the pandemic.

#### Immune function analysis

The cellular immune function, as indicated by the lymphocyte counts of CD3^+^, CD4^+^, and CD8^+^, was significantly reduced after the pandemic compared to the same period before and during the pandemic (*P* < 0.001) (**Table 3**). Conversely, the humoral immune function, as evidenced by the levels of IgG, IgA, IgM, and complement C3, was significantly higher after the pandemic than before and during the pandemic (*P* < 0.001).

**Table 3:** Analysis of immune function in the study population*

Characteristics	Before the pandemic	During the pandemic	After the pandemic	*P*-value†
IgG, g/L	7.26 (5.73, 9.51)	6.79 (4.95, 9.08)	8.24 (6.26, 10.38)§	<0.001
IgA, g/L	0.64 (0.27, 1.19)	0.51 (0.28, 0.95)‡	0.80 (0.36, 1.35)§	<0.001
IgM, g/L	1.12 (0.73, 1.56)	0.95 (0.66, 1.27)‡	1.13 (0.79, 1.51)¶	<0.001
IgE, IU/mL	90.27 (37.00, 242.00)	83.30 (21.80, 356.50)	93.10 (31.60, 292.00)	0.768
Complement C3, g/L	1.08 (0.89, 1.31)	1.05 (0.87, 1.22)	1.13 (0.97, 1.36)§	<0.001
Complement C4, g/L	0.25 (0.19, 0.34)	0.26 (0.20, 0.32)	0.26 (0.20, 0.31)	0.921
CD3^+^, cells/μL	2700 (1674, 4380)	2784 (1835, 4019)	1342 (847, 2076)§	<0.001
CD4^+^, cells/μL	1456 (900, 2440)	1524 (1004, 2412)	752 (463, 1318)§	<0.001
CD8^+^, cells/μL	1020 (612, 1696)	1002 (615, 1444)	494 (289, 777)§	<0.001
CD19^+^, cells/μL	1102 (418, 1786)	1260 (689, 1545)	830 (365, 1295)	0.561

IQR – interquartile range, MD – median

*Presented as MD (IQR).

†Pearson’s χ^2^ test.

‡*P* < 0.05, compared with before the pandemic.

§*P* < 0.05, compared with before and during the pandemic.

¶*P* < 0.05, compared with during the pandemic.

**Correspondence to:** Bing He Renmin Hospital of Wuhan University, Department of Pediatrics No. 238 Jiefang Road, Wuhan 430060, Hubei Province China rm001144@whu.edu.cn

#### Pathogen detection rate before, during, and after the pandemic

We examined a total of 5180 children with CAP for pathogens, identifying at least one in 2566 children (49.5%). The proportion of bacterial infections before the pandemic (4.9%) was significantly lower than during (9.7%) and after the pandemic (9.7%) (*P* < 0.001) (Table S1 in the **Online Supplementary Document****)**. In contrast, the proportion of virus (20.1%) and *MP* infections (41.9%) after the pandemic was significantly higher than before (7.4% and 36.3%) and during the pandemic (9.0% and 18.3%) (*P* < 0.001).

#### Pathogen composition before, during, and after the pandemic

**Figure 2:**
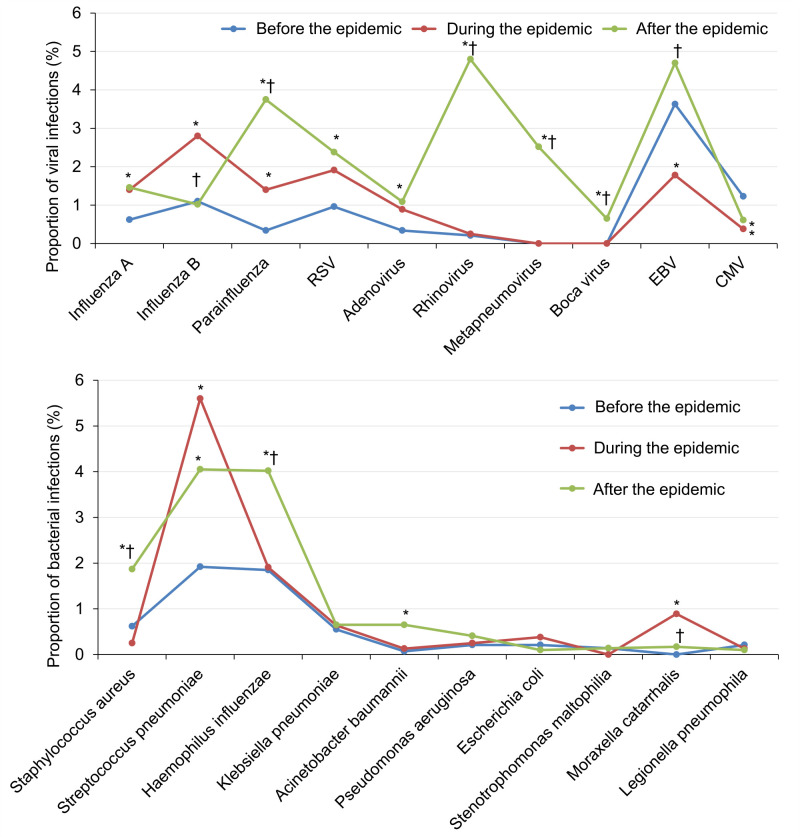
Viral and bacterial infections of children with CAP before, during, and after the pandemic. *P < 0.05 *vs.* before the pandemic. †*P* < 0.05 *vs*. during the pandemic. CAP – community-acquired pneumonia.

The positive rates of rhinovirus, parainfluenza, and metapneumovirus showed the most significant increases after the pandemic compared to the pre-pandemic and pandemic periods. Additionally, the detection rates of *Haemophilus influenzae* and *Staphylococcus aureus* after the pandemic were significantly higher than before and during the pandemic, while the detection rate of *Streptococcus pneumoniae* was highest during the pandemic (**Figure 2**).

#### Pathogen composition in children with CAP of different age groups

**Figure 3:**
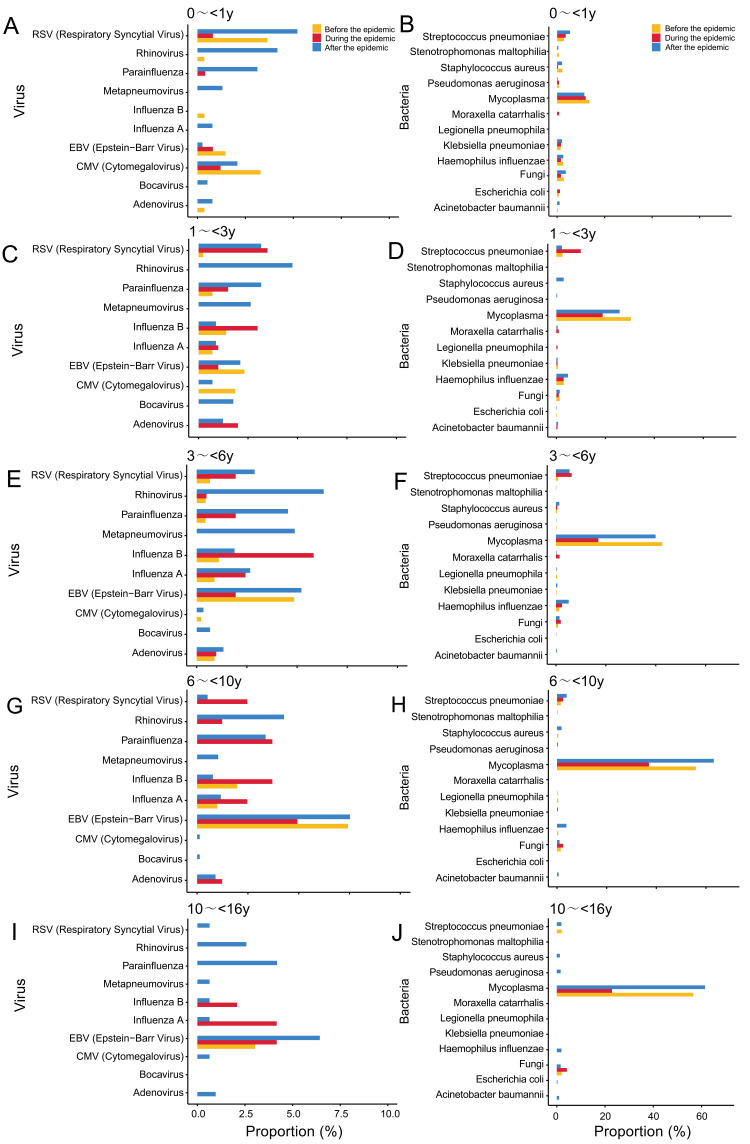
Age-stratified visualisations of pathogen distribution trends. **Panel A.** Distribution of viral infection in CAP children aged 0–1 year. **Panel B.** Distribution of bacterial infection in CAP children aged 0–1 year. **Panel C.** Distribution of viral infection in CAP children aged 1–3 years. **Panel D.** Distribution of bacterial infection in CAP children aged 1–3 years. **Panel E.** Distribution of viral infection in CAP children aged 3–6 years. **Panel F.** Distribution of bacterial infection in CAP children aged 3–6 years. **Panel G.** Distribution of viral infection in CAP children aged 6–10 years. **Panel H.** Distribution of bacterial infection in CAP children aged 6–10 years. **Panel I.** Distribution of viral infection in CAP children aged 10–16 years. **Panel J.** Distribution of bacterial infection in CAP children aged 10–16 years. CAP – community-acquired pneumonia.

The virus detection rate was lowest in infants <1 year of age (9.9%) (**Figure 3**, Panel A) among preschool children when compared to other age groups, whereas the virus infection rate was highest (18.9%) (**Figure 3**, Panel E). The predominant viruses identified were EBV, rhinovirus, influenza virus, parainfluenza virus, and RSV. The detection rates for rhinovirus, influenza virus, and parainfluenza virus were highest in children aged 3–6 years (**Figure 3**, Panel E). We observed the highest rate of RSV positivity in infants <1 year of age (3.6%) (**Figure 3**, Panel A), while we found the greatest incidence of EBV infection in children aged 6–10 years (7.4%) (**Figure 3**, Panel G).

We found the highest bacterial detection rate (11.0%) in infants <1 year old. The bacterial detection rate exhibited a gradual decline with increasing age. The most commonly identified bacteria included *Streptococcus pneumoniae*, *Haemophilus influenzae*, and *Staphylococcus aureus*. The positivity rate for *Staphylococcus aureus* was highest among infants <1 year old (1.7%) (**Figure 3**, Panel B), while we observed the highest detection rate of *Streptococcus pneumoniae* in children aged 3–6 years at 4.2% (**Figure 3**, Panel F). We found the highest positivity rate for *Haemophilus influenzae* in children aged 1–3 years (3.8%) (**Figure 3**, Panel D).

Children aged 6–10 and 10–16 years showed the highest detection rates of *MP*, at 59.8% and 56.3%, respectively (**Figures 3**, Panels H and J). Conversely, the detection rate of *MP* decreased gradually with a decrease in age. Specifically, the detection rates were 37.7% (**Figure 3**, Panel F) in preschool children aged 3–6 years, 26.3% in children aged 1–3 years (**Figure 3**, Panel D), and 12.2% in infants aged <1 year (**Figure 3**, Panel B). In contrast, infants <1 year old (2.9%) exhibited the highest fungal detection rate (**Figure 3**, Panel B), while children aged 6–10 years had the lowest fungal infection rate of 1.2% (**Figure 3**, Panel H).

#### Rate of pathogenic coinfection in children with CAP across different ages and before, during, and after the pandemic

The rates of viral co-infection (3.1%), viral-bacterial co-infection (3.3%), and bacterial co-infection (1.8%) among children aged 3–6 years were higher than those observed in other age groups (Figures S1 and S2 in the **Online Supplementary Document**). The rates of viral-*MP* co-infection (10.5%) and bacterial-*MP* co-infection (4.6%) in children aged 6–10 years were also higher than those in other age categories.

Looking at the three different periods, the rate of co-infection in children with CAP after the pandemic was significantly higher than before and during the pandemic (Figures S1 and S2 in the **Online Supplementary Document**). Specifically, the rates of viral co-infection (2.7%), viral-bacterial coinfection (4.0%), bacterial co-infection (2.1%), viral-*MP* coinfection (9.0%), and bacterial-*MP* coinfection (5.0%) were all increased after the pandemic compared to before and during the pandemic.

#### Composition of pathogenic co-infections in CAP children

The highest rate of virus-virus co-infection was observed with influenza A and influenza B (0.71%). The highest rate of bacteria-bacteria co-infection was recorded for *Streptococcus pneumoniae* and *Haemophilus influenzae* (0.64%), while the highest virus-bacteria co-infection was *Haemophilus influenzae* and rhinovirus (0.35%). Furthermore, the highest proportion of *MP*-bacteria co-infection was between *MP* and *Streptococcus pneumoniae* (1.43%), and the highest *MP*-virus coinfection was found between *MP* and EBV (2.32%) (Figure S3 in the **Online Supplementary Document**).

### Identification of independent risk factors for SCAP

#### Comparison of clinical symptoms and pathogens between <8 and ≥8 CT score groups

Among the 5180 children with CAP, 2729 underwent CT examinations. They were categorised into the CT score <8 group (n = 2440) and the CT ≥8 group (n = 289). The latter showed significantly higher proportions of allergic constitution (*P* = 0.028), fever (*P* = 0.003), dyspnoea (*P* = 0.001), wheezing (*P* = 0.002), thoracodynia (*P* < 0.001), lung consolidation (*P* < 0.001), and complications such as myocardial injury (*P* < 0.001), liver function impairment (*P* < 0.001), coagulation dysfunction (*P* < 0.016), and bronchiolitis obliterans (*P* = 0.001) (Table S2 in the **Online Supplementary Document**). This group also had a greater median duration of fever (*P* = 0.008) and hospital stay (*P* < 0.001) compared to the CT score <8 group. However, the incidence of cough was significantly lower in the CT score ≥8 group than in the CT score <8 group (*P* = 0.001).

The proportions of *Klebsiella pneumoniae* (*P* = 0.002), *Acinetobacter baumannii* (*P* = 0.022), and adenovirus (*P* = 0.016) in the CT score ≥8 group were significantly higher than those in the CT score <8 group, while the proportions of *Haemophilus influenzae* (*P* = 0.041) and parainfluenza (*P* = 0.019) were significantly lower (Table S3 in the **Online Supplementary Document**).

#### Independent risk factors for SCAP

Multivariate Cox regression analysis identified the following independent risk factors for SCAP: wheezing (hazard ratio (HR) = 1.551; 95% CI = 1.172, 2.053), chest pain (HR = 3.144; 95% CI = 1.922, 5.143), haemoptysis (HR = 4.854; 95% CI = 1.205, 19.551), *Klebsiella pneumoniae* (HR = 4.236; 95% CI = 1.870, 9.596), *Acinetobacter baumannii* (HR = 4.432; 95% CI = 1.650, 11.907), influenza B (HR = 2.338; 95% CI = 1.040, 5.257), and adenovirus (HR = 2.895; 95% CI = 1.365, 6.140) (Figure S4 in the **Online Supplementary Document**). The identified risk factors remained unchanged after adjustments for confounding, suggesting the findings to be robust. The proportional hazards assumption was met based on the Kaplan-Meier survival analysis (data not shown). The sensitivity analysis further confirmed the robustness of the identified risk factors (data not shown).

The area under the receiver operating characteristics was 0.797 (95% CI = 0.758–0.837) (Figure S4 in the **Online Supplementary Document**), suggesting that our risk factor model has good predictive performance.

## DISCUSSION

CAP is a significant health threat to children worldwide, ranking as the leading cause of death among children under five years of age in low- and middle-income countries [18]. Here we explored the aetiology and epidemiological characteristics of CAP in children across 13 provinces of China. We examined variations in pathogen infections and co-infection patterns across different age groups and before, during, and after the COVID-19 pandemic, while also determining independent risk factors for SCAP. While SARS-CoV-2 emerged as a notable pathogen responsible for CAP in children from 2020 to 2022, we did not include children with COVID-19 in our analysis, as the hospitals involved in our study were not designated for such admissions according to the Chinese pandemic prevention and control protocols.

The prolonged implementation of NPIs during the COVID-19 pandemic has significantly decreased infections from other pathogens globally [19], creating a so-called ‛immunity debt’ that could lead to widespread respiratory pathogen outbreaks. Moreover, the persistent presence of SARS-CoV-2 in affected individuals’ bodies, commonly referred to as long COVID-19, may have long-term effects on their immune function, potentially leading to the activation of latent viruses [20]. This condition can also result in tissue damage and dysregulation of the coagulation system. The impact of interruptions in regular vaccination campaigns must be considered in this context, given the increases in global measles cases and regional outbreaks since 2022 [20]. Researchers have further suggested that under specific circumstances, where the prevalence of other pathogens is inhibited by the long-term effects of the COVID-19 pandemic and NPIs, adaptive changes may occur in other microbial pathogens [21]. As NPIs were lifted, China experienced peaks in infections from influenza virus, RSV, rhinovirus, parainfluenza virus, and *MP* in 2023 [22]. Surveillance data from Melbourne, Australia showed that NPIs reduced the proportions of children testing positive for influenza A, influenza B, and RSV in Melbourne, Australia [23]. However, after NPIs were lifted, RSV infections among Australian children resurged in the interseasonal period [24]. Similarly, a population-based national cohort study in Denmark showed a threefold increase in *MP* infections and hospitalisations in 2023–24 compared to the pre-COVID-19 period [25]. Wang and colleagues found that during the 2022–23 influenza season, the peak incidence of influenza infections in both southern and northern China and the USA was 2.5–3 times higher than the levels recorded between 2011 and 2019 [26]. Our findings on the trends in CAP align with these studies, emphasising the significant shifts in respiratory pathogen trends worldwide and pointing to a need for ongoing monitoring and research to understand the long-term effects of pandemic-related interventions on CAP and other respiratory infections.

A nationwide study in China reported an overall CAP incidence rate of 7.13 cases per 1000 person-years, primarily affecting children under five years old and elderly individuals aged 80 years and older [27]. The incidence rates were 7.32 cases per 1000 person-years for males and 6.93 cases per 1000 person-years for females. Consistently, we found that infants and preschool children constituted the majority of hospitalised cases of CAP, with incidence decreasing as age increased, meaning that children of a younger age were at greater risk for developing CAP. However, the proportion of children over six years old diagnosed with CAP has increased in 2023 [11]. This could be largely attributable to the prevalence of *MP* after the pandemic, since it has been more commonly observed in school-age children and adolescents [28].

Fever, cough, and wheezing are critical clinical manifestations of CAP in children [29]. We found that fever was more prevalent among children diagnosed with CAP after the pandemic, while those with SCAP experienced longer durations of fever. This could be related to the higher incidence of CAP in older children after the pandemic, whose immune systems are more fully developed, leading to a more pronounced systemic inflammatory response post-infection. Hence, the frequency and duration of fever episodes were both elevated in this age group. Moreover, our results also indicate that wheezing, chest pain, and haemoptysis were characteristic features of SCAP. Wheezing is typically associated with viral or *MP* infections. We arrived at a conclusion similar to that of Chen and colleagues, who identified wheezing as an independent risk factor for SCAP or transfer to the intensive care unit in children over two years old [30]. The 2019 guidelines for CAP in children [29] further highlight that an increased respiratory rate is a significant indicator of CAP severity in children. Chest pain, often resulting from an extensive lung infection that involves the pleura, pleurisy, or pleural effusion, is another common symptom of CAP in this population. Additionally, the occurrence of haemoptysis warrants consideration of SCAP, particularly in cases linked to multiple lobar infiltrations of *Staphylococcus aureus* [31]. Therefore, if a child exhibits these symptoms, the possibility of SCAP should be thoroughly evaluated.

We found that children with CAP had low cellular immune function and decreased T lymphocytes after the pandemic. This may be similar to the extensive T lymphocytopenia and immune dysregulation experienced by many children infected with COVID-19 after the lifting of NPIs in China in late 2022, with levels returning to their baseline 6–12 months after infection [32]. T lymphocytes are crucial for the cellular immune response *in vivo* and are key indicators of the body’s immune state, playing an important role in acquired immune responses to infections [33]. Children with weakened cellular immune function are more prone to multiple pathogen infections, increasing the risk of SCAP [34], which we also evident in our observations. Notably, the humoral immune function of children with CAP after the pandemic was higher compared to before and during the pandemic, potentially attributed to a predominance of older children over the age of six among those with CAP after the pandemic. It is well-established that levels of immunoglobulins IgG, IgA, and IgM increase with age, reaching adult levels by around 10 years old, suggesting that humoral immunity levels in children with CAP after the pandemic are elevated [35].

The pathogens associated with CAP primarily include viruses, bacteria, and *MP*. We found *MP* to be the most commonly detected pathogen in children with CAP after the pandemic, consistent with findings that it has increasingly become the primary pathogenic agent of CAP in children [36]. Though *MP* infections can occur across all age groups, they are predominantly observed in children over six years old, while infection rates show an upward trend with advancing age [37], which is consistent with our results. Yan and colleagues indicate that the dominant genotypes of *MP* in Japan and northern China have shifted from type P1 to type P2, and from genotype M4-5-7-2 to genotype M3-5-6-2 [6]. Additionally, the M3-5-6-2 genotype is associated with severe *MP*, with the resistance rate of M3-5-6-2 strains to macrolide antibiotics increasing from 60% to 93.48% [38,39]. Whether the current outbreak of *MP* is linked to subtype variations of the dominant circulating strains remains to be investigated.

Roh and colleagues identified viral infections as the most prevalent cause of CAP in children under five years of age, with infection rates decreasing as age increases [40]. We obtained similar results, indicating that young children are particularly susceptible to viral infections. Rueda and colleagues found that the most common viruses that may cause CAP in children include RSV, influenza virus, parainfluenza virus, rhinovirus, and adenovirus [41], which is similar to our results. The COVID-19 pandemic has significantly impacted the epidemiological patterns of viral transmissions [19]. For example, the occupancy effect of SARS-CoV-2 can influence the interactions between various viruses and their hosts, each with distinct characteristics [42]. Interestingly, the detection rates of viral infections in children significantly increased after compared to before and during the pandemic. This increase may be attributed to their generally diminished cellular immune function after the pandemic, alongside a surge in multiple viral infections after the lifting of NPIs in China [32]. In contrast to the high detection rates of influenza virus and RSV before and during the pandemic, the rhinovirus, EBV, and parainfluenza virus displayed the highest detection rates in our study after the pandemic, with rhinovirus and parainfluenza virus showing the largest increases. Studies have shown rhinovirus to be the second most common virus cause of CAP globally, with a detection rate of 22.1%, surpassed only by RSV at 22.7% [43,44]. Furthermore, we observed a significant prevalence of EBV in children diagnosed with CAP, with a higher incidence in older children. EBV is a globally prevalent herpesvirus whose infections remain asymptomatic and latent, suggesting that it may serve as a non-pathogenic agent detected in older children. Röltgen and colleagues propose that the persistent presence of SARS-CoV-2 in the host may potentially activate other latent viruses, such as EBV [45]. This underscores the importance of conducting EBV testing in clinical practice, particularly among older children with CAP, as it may assist in evaluating disease progression. Additionally, the detection rate of parainfluenza virus was found to be the highest in children under five years [46,47], which aligns with our findings. The detection rate of the influenza virus in our study did not significantly increase after the pandemic compared to before and during the pandemic. This is likely because the COVID-19 pandemic disrupted the seasonality of influenza, shifting its peak to an earlier period (February to April 2023) [48]. Our study was, meanwhile, conducted outside this shifted peak season.

Bacteria are prevalent pathogens associated with CAP in children. The primary pathogens responsible for CAP include *Streptococcus pneumoniae*, *Haemophilus influenzae*, and *Staphylococcus aureus* [29], which is consistent with our findings. The detection rates of bacteria were the lowest before the pandemic, increasing during and after the outbreak to a similar extent. Moreover, the proportion of bacterial infections during the pandemic was the highest among infants and young children in our study. This increase can be attributed to the fact that the majority of hospitalised children with CAP were infants and young children. Additionally, after the pandemic, diminished cellular immune function made children more susceptible to co-infections with *MP* and bacteria, resulting in higher bacterial detection rates during and after compared to before the pandemic. Our results also align with studies that have reported *Haemophilus influenzae* as the most common infection in children aged 1–3 years and the one with the highest detection rates [49,50]. *Staphylococcus aureus* is widely prevalent in the environment and is a significant pathogen associated with CAP, with the highest detection rates found in infants <1 year old [51,52], which is consistent with our results. Considering that early childhood is the typical age for starting daycare, the immune systems of infants at this stage may not be fully developed, making them more vulnerable to pathogens, which is consistent with higher carrier rates of *Streptococcus pneumoniae* in this age group [53]. This observation aligns with findings reported by Guo and colleagues [54] regarding increased *Streptococcus pneumoniae* positivity rates during the pandemic.

The rate of multiple pathogen co-infections in children with CAP was higher after than before and during the pandemic. This increase may be attributed to the generally compromised cellular immune function observed in children after the pandemic, which renders mixed infections with multiple pathogens more likely [32]. These results suggest that the aetiology of CAP in children presents an age-dependent pattern, and that the pathogen spectrum of CAP has correspondingly evolved following the COVID-19 outbreak.

SCAP is characterised by the rapid onset of inflammation affecting one or more lung lobes, which may lead to various complications and pose a significant threat to life [15]. We found that *Klebsiella pneumoniae*, *Acinetobacter baumannii*, influenza B, and adenovirus were pathogenic risk factors associated with SCAP. The former two are gram-negative opportunistic pathogens that exhibit rising resistance rates to carbapenems; they are particularly adept at infecting children with pre-existing conditions and compromised immune systems [55,56], leading to community-acquired lobar pneumonia [57]. *Acinetobacter baumannii* has also been implicated in the development of SCAP [58], primarily affecting individuals with underlying health issues, thereby increasing patient mortality. We found that 64% of children with CAP caused by *Klebsiella pneumoniae* and *Acinetobacter baumannii* were under two years of age (data not shown), reflecting their low immune function. Additionally, the mortality rate for children with influenza B virus pneumonia admitted to intensive care units is approximately 25% [59], indicating that they face an increased risk of severe disease and mortality, a matter deserving of clinical attention. Furthermore, recent reports note that the incidence of adenovirus pneumonia in children ranges from 4% to 10%, accounting for about 11.9% of SCAP cases in this demographic [60,61]. Therefore, the clinical identification of infections needs careful consideration by healthcare professionals.

Considering our study’s findings and the changing dynamics of infectious diseases post-pandemic, we recommend the following policies to improve the prevention and management of CAP in children. First, we propose the strengthening of national and regional surveillance systems to monitor trends in CAP incidence, pathogen distribution, and antibiotic resistance patterns to facilitate timely public health responses. Second, an expansion of vaccination programmes could increase access to vaccines targeting key CAP pathogens, such as *Streptococcus pneumoniae* and *Haemophilus influenzae*, while raising awareness of the importance of improving vaccine coverage in the paediatric population. Third, we propose the implementation of infection control practices in schools, childcare centres, and healthcare facilities to promote hygiene measures such as handwashing and respiratory etiquette. Additionally, the launch of public awareness campaigns could educate caregivers and healthcare providers on the signs and symptoms of CAP, the importance of its early recognition, and when to seek medical attention. Ensuring timely access to healthcare services could help children during peak respiratory infection seasons, facilitating early diagnosis and treatment to reduce complications. Research and development of novel vaccines and treatments for CAP should also be encouraged, particularly for emerging pathogens and antibiotic-resistant strains. Finally, fostering collaboration among government agencies, healthcare organisations, and educational institutions could help develop coordinated prevention and management strategies.

### Limitations

We gathered data on CAP cases from August to October during 2018, 2020, and 2023, thereby introducing seasonal biases. Future studies could investigate pathogen distributions across different seasons to provide a comprehensive understanding of CAP dynamics. Furthermore, we obtained data from hospitals in 13 distinct regions, so the variations in climate, environment, and sensitivity of pathogen detection methods may result in differences in pathogen composition. We also focussed exclusively on hospitalised children with CAP, excluding outpatient cases and children with asthma or chronic illnesses, which may lead to potential underreporting biases and introduce potential selection bias, affecting the generalisability of our findings. Moreover, we did not collect data on the impact of vaccination coverage (influenza, pneumococcal, and RSV vaccines) on CAP trends. Future research should prioritise the evaluation of how variations in vaccination coverage influence the trends of CAP. Lastly, bacterial colonisation in children’s airways, coupled with the risk of contamination in respiratory tract specimens, may compromise the accuracy of pathogen detection. Future multi-centre studies are warranted to include a range of respiratory specimens and diverse detection techniques to improve the accuracy of pathogen identification and to clarify whether detected organisms are pathogenic or merely colonisers.

## CONCLUSIONS

In our analysis of children with CAP across 13 Chinese provinces before, during, and after the COVID-19 pandemic, we detected shifts in pathogen prevalence and immune function, highlighting the lasting effects of the pandemic on children’s health. Moreover, we observed a marked increase in the incidence of SCAP after the pandemic, particularly among older children, along with a significant rise in viral and *MP* infections. Furthermore, we identified several independent risk factors for SCAP, including specific pathogens and clinical manifestations such as wheezing and chest pain. These findings provide a scientific basis for the diagnosis, differential diagnosis, and rational use of antibiotics for CAP post-COVID-19. They also highlight the importance of recognising pneumonia risk factors and implementing timely interventions and treatments to reduce the incidence of SCAP among high-risk populations.

## Additional material


Online Supplementary Document

## References

[1] Liu Y Morgenstern C Kelly J Lowe R Group CC-W, Jit M. The impact of non-pharmaceutical interventions on SARS-CoV-2 transmission across 130 countries and territories. BMC Med. 2021;19:40. 10.1186/s12916-020-01872-8 33541353 PMC7861967

[2] Yang DD Ouldali N Gajdos V Thomas-Sertillanges R Vasante L Skurnik D Common Pediatric Respiratory Infectious Diseases as Possible Early Predictor for New Wave of Severe Acute Respiratory Syndrome Coronavirus 2 Infections. Clin Infect Dis. 2021;73:358–9. 10.1093/cid/ciaa1359 32894752 PMC7499521

[3] Vaux S Viriot D Forgeot C Pontais I Savitch Y Barondeau-Leuret A Bronchiolitis epidemics in France during the SARS-CoV-2 pandemic: The 2020-2021 and 2021-2022 seasons. Infect Dis Now. 2022;52:374–8. 10.1016/j.idnow.2022.06.003 35753628 PMC9222408

[4] Mondal P Sinharoy A Gope S The Influence of COVID-19 on Influenza and Respiratory Syncytial Virus Activities. Infect Dis Rep. 2022;14:134–41. 10.3390/idr14010017 35200444 PMC8872472

[5] Zhang XB He W Gui YH Lu Q Yin Y Zhang JH Current Mycoplasma pneumoniae epidemic among children in Shanghai: unusual pneumonia caused by usual pathogen. World J Pediatr. 2024;20:5–10. 10.1007/s12519-023-00793-9 38231466

[6] Yan C Xue GH Zhao HQ Feng YL Cui JH Yuan J Current status of Mycoplasma pneumoniae infection in China. World J Pediatr. 2024;20:1–4. 10.1007/s12519-023-00783-x 38185707 PMC10827902

[7] Pezzotti P Bellino S Prestinaci F Iacchini S Lucaroni F Camoni L The impact of immunization programs on 10 vaccine preventable diseases in Italy: 1900-2015. Vaccine. 2018;36:1435–43. 10.1016/j.vaccine.2018.01.065 29428176

[8] Ho LL Gurung S Mirza I Nicolas HD Steulet C Burman AL Impact of the SARS-CoV-2 pandemic on vaccine-preventable disease campaigns. Int J Infect Dis. 2022;119:201–9. 10.1016/j.ijid.2022.04.005 35398300 PMC8985404

[9] Posten S Reed J Pediatric Community Acquired Pneumonia. S D Med. 2017;70:557–61. 29334446

[10] McAllister DA Liu L Shi T Chu Y Reed C Burrows J Global, regional, and national estimates of pneumonia morbidity and mortality in children younger than 5 years between 2000 and 2015: a systematic analysis. Lancet Glob Health. 2019;7:e47–57. 10.1016/S2214-109X(18)30408-X 30497986 PMC6293057

[11] Zhang XB He W Gui YH Lu Q Yin Y Zhang JH Current Mycoplasma pneumoniae epidemic among children in Shanghai: unusual pneumonia caused by usual pathogen. World J Pediatr. 2024;20:5–10. 10.1007/s12519-023-00793-9 38231466

[12] Liu YN Zhang YF Xu Q Qiu Y Lu QB Wang T Infection and co-infection patterns of community-acquired pneumonia in patients of different ages in China from 2009 to 2020: a national surveillance study. Lancet Microbe. 2023;4:e330–9. 10.1016/S2666-5247(23)00031-9 37001538 PMC12514336

[13] World Health Organization. Pocket Book of Hospital Care for Children: Guidelines for the Management of Common Childhood Illnesses. Geneva, Switzerland: World Health Organization; 2013. Available: https://www.who.int/publications/i/item/978-92-4-154837-3. Accessed: 2 July 2025. 24006557

[14] Li K Fang Y Li W Pan C Qin P Zhong Y CT image visual quantitative evaluation and clinical classification of coronavirus disease (COVID-19). Eur Radiol. 2020;30:4407–16. 10.1007/s00330-020-06817-6 32215691 PMC7095246

[15] Subspecialty Group of Respiratory tSoP Chinese Medical Association Editorial Board CJoP China Medicine Education Association Committee on Pediatrics [Guidelines for the management of community-acquired pneumonia in children (2024 revision)]. Zhonghua Er Ke Za Zhi. 2024;62:920–30. Chinese. 39327958 10.3760/cma.j.cn112140-20240728-00523

[16] Moyano A Ferressini N De Matteo E Preciado MV Chabay P PD-L1 is upregulated in CD163^+^ tonsillar macrophages from children undergoing EBV primary infection. Front Immunol. 2022;13:940910. 10.3389/fimmu.2022.940910 36451810 PMC9701750

[17] Gaur AS, Gaur SS. Statistical Methods for Practice and Research: A Guide to Data Analysis Using SPSS. 2nd ed. New Delhi, India: Sage Publication; 2006

[18] Rodrigues CMC Groves H Community-Acquired Pneumonia in Children: the Challenges of Microbiological Diagnosis. J Clin Microbiol. 2018;56:e01318–17. 10.1128/JCM.01318-17 29237789 PMC5824044

[19] Yang MC Su YT Chen PH Tsai CC Lin TI Wu JR Changing patterns of infectious diseases in children during the COVID-19 pandemic. Front Cell Infect Microbiol. 2023;13:1200617. 10.3389/fcimb.2023.1200617 37457965 PMC10339349

[20] Burrell R Saravanos G Britton PN Unintended impacts of COVID-19 on the epidemiology and burden of paediatric respiratory infections. Paediatr Respir Rev. 2025;53:3–13. 10.1016/j.prrv.2023.07.004 37580220

[21] Fang F Reflections on the trends of infectious diseases in children after the SARS-CoV-2 pandemic. Zhonghua Er Ke Za Zhi. 2024;62:703–5. 39039870 10.3760/cma.j.cn112140-20240610-00388

[22] Chi J Tang H Wang F Wang Y Chen Z Surge in Mycoplasma Pneumoniae infection and Respiratory Viruses Co-infection in Children With Community-Acquired Pneumonia in the Post-Pandemic. Pediatric Health Med Ther. 2024;15:279-88. 10.2147/PHMT.S473669 39263589 PMC11389692

[23] Abo YN Clifford V Lee LY Costa AM Crawford N Wurzel D COVID-19 public health measures and respiratory viruses in children in Melbourne. J Paediatr Child Health. 2021;57:1886–92. 10.1111/jpc.15601 34080245 PMC8242487

[24] Foley DA Yeoh DK Minney-Smith CA Martin AC Mace AO Sikazwe CT The Interseasonal Resurgence of Respiratory Syncytial Virus in Australian Children Following the Reduction of Coronavirus Disease 2019-Related Public Health Measures. Clin Infect Dis. 2021;73:e2829–30. 10.1093/cid/ciaa1906 33594407 PMC7929151

[25] Dungu KHS Holm M Hartling U Jensen LH Nielsen AB Schmidt LS *Mycoplasma pneumoniae* incidence, phenotype, and severity in children and adolescents in Denmark before, during, and after the COVID-19 pandemic: a nationwide multicentre population-based cohort study. Lancet Reg Health Eur. 2024;47:101103. 10.1016/j.lanepe.2024.101103 39469091 PMC11513821

[26] Wang Q Jia M Jiang M Cao Y Dai P Yang J Increased population susceptibility to seasonal influenza during the COVID-19 pandemic in China and the United States. J Med Virol. 2023;95:e29186. 10.1002/jmv.29186 37855656

[27] Sun Y Li H Pei Z Wang S Feng J Xu L Incidence of community-acquired pneumonia in urban China: A national population-based study. Vaccine. 2020;38:8362–70. 10.1016/j.vaccine.2020.11.004 33199077

[28] Li J Tang W Wang W Application value of erythromycin combined with azithromycin sequential therapy in the treatment of mycoplasma pneumonia in children. Minerva Pediatr (Torino). 2022;74:247–9. 10.23736/S2724-5276.21.06518-6 34264050

[29] Subspecialty Group of Respiratory Diseases TSoP Chinese Medical Association, Editorial Board CJoP [Guidelines for management of community acquired pneumonia in children (the revised edition of 2013) (I)]. Zhonghua Er Ke Za Zhi. 2013;51:745–52. 24406226

[30] Chen L Miao C Chen Y Han X Lin Z Ye H Age-specific risk factors of severe pneumonia among pediatric patients hospitalized with community-acquired pneumonia. Ital J Pediatr. 2021;47:100. 10.1186/s13052-021-01042-3 33892752 PMC8062938

[31] He H Wunderink RG Staphylococcus aureus Pneumonia in the Community. Semin Respir Crit Care Med. 2020;41:470–9. 10.1055/s-0040-1709992 32521547

[32] Taeschler P Adamo S Deng Y Cervia C Zurbuchen Y Chevrier S T-cell recovery and evidence of persistent immune activation 12 months after severe COVID-19. Allergy. 2022;77:2468–81. 10.1111/all.15372 35567391 PMC9347640

[33] Kruglova TS Fomina DS The informative value of CD3+CD4+ and CD3+CD8+ T-cell count and cHIS scale as predictors of severe COVID-19 when using interleukin-6 receptor blockers in the in-hospital setting. Ter Arkh. 2022;94:1294–302. 10.26442/00403660.2022.11.202002 37167168

[34] Geng B Ding X Li X Liu H Zhao W Gong H Peripheral blood T-lymphocyte subsets are potential biomarkers of disease severity and clinical outcomes in patients with ulcerative colitis: a retrospective study. BMC Gastroenterol. 2023;23:136. 10.1186/s12876-023-02769-5 37106335 PMC10134527

[35] Bayram RO Ozdemir H Emsen A Turk Dagi H Artac H Reference ranges for serum immunoglobulin (IgG, IgA, and IgM) and IgG subclass levels in healthy children. Turk J Med Sci. 2019;49:497–505. 10.3906/sag-1807-282 30997788 PMC7018341

[36] Greenberg D Givon-Lavi N Faingelernt Y Ben-Shimol S Avni YS Bar-Ziv J Nasopharyngeal Pneumococcal Carriage During Childhood Community-Acquired Alveolar Pneumonia: Relationship Between Specific Serotypes and Coinfecting Viruses. J Infect Dis. 2017;215:1111–6. 28011920 10.1093/infdis/jiw613

[37] Cheng Y Cheng Y Dai S Hou D Ge M Zhang Y The Prevalence of Mycoplasma Pneumoniae Among Children in Beijing Before and During the COVID-19 Pandemic. Front Cell Infect Microbiol. 2022;12:854505. 10.3389/fcimb.2022.854505 35573799 PMC9103471

[38] Yan C Xue G Zhao H Feng Y Li S Cui J Molecular and clinical characteristics of severe Mycoplasma pneumoniae pneumonia in children. Pediatr Pulmonol. 2019;54:1012–21. 10.1002/ppul.24327 31119869

[39] Wang Y Xu B Wu X Yin Q Wang Y Li J Increased Macrolide Resistance Rate of M3562 Mycoplasma pneumoniae Correlated With Macrolide Usage and Genotype Shifting. Front Cell Infect Microbiol. 2021;11:675466. 10.3389/fcimb.2021.675466 34055671 PMC8149950

[40] Roh EJ Shim JY Chung EH Epidemiology and surveillance implications of community-acquired pneumonia in children. Clin Exp Pediatr. 2022;65:563–73. 10.3345/cep.2022.00374 36265520 PMC9742763

[41] Rueda ZV Aguilar Y Maya MA Lopez L Restrepo A Garces C Etiology and the challenge of diagnostic testing of community-acquired pneumonia in children and adolescents. BMC Pediatr. 2022;22:169. 10.1186/s12887-022-03235-z 35361166 PMC8968093

[42] Muangnoicharoen S Wiangcharoen R Lawpoolsri S Nanthapisal S Jongkaewwattana A Duangdee C Heterologous Ad26.COV2.S booster after primary BBIBP-CorV vaccination against SARS-CoV-2 infection: 1-year follow-up of a phase 1/2 open-label trial. Vaccine. 2024;42:3999–4010. 10.1016/j.vaccine.2024.05.010 38744598

[43] Pratt MTG Abdalla T Richmond PC Moore HC Snelling TL Blyth CC Prevalence of respiratory viruses in community-acquired pneumonia in children: a systematic review and meta-analysis. Lancet Child Adolesc Health. 2022;6:555–70. 10.1016/S2352-4642(22)00092-X 35636455

[44] Zhu Y Xu B Li C Chen Z Cao L Fu Z A Multicenter Study of Viral Aetiology of Community-Acquired Pneumonia in Hospitalized Children in Chinese Mainland. Virol Sin. 2021;36:1543–53. 10.1007/s12250-021-00437-0 34523109 PMC8440149

[45] Röltgen K Boyd SD Antibody and B Cell Responses to SARS-CoV-2 Infection and Vaccination: The End of the Beginning. Annu Rev Pathol. 2024;19:69–97. 10.1146/annurev-pathmechdis-031521-042754 37738512

[46] Pan Y Zhang Y Shi W Peng X Cui S Zhang D Human parainfluenza virus infection in severe acute respiratory infection cases in Beijing, 2014-2016: A molecular epidemiological study. Influenza Other Respir Viruses. 2017;11:564–8. 10.1111/irv.12514 29054112 PMC5705688

[47] Chen Q Lin L Zhang N Yang Y Adenovirus and Mycoplasma pneumoniae co-infection as a risk factor for severe community-acquired pneumonia in children. Front Pediatr. 2024;12:1337786. 10.3389/fped.2024.1337786 38357505 PMC10864498

[48] Zheng L Lin Y Yang J Fang K Wu J Zheng M Global variability of influenza activity and virus subtype circulation from 2011 to 2023. BMJ Open Respir Res. 2023;10:e001638. 10.1136/bmjresp-2023-001638 37491131 PMC10577751

[49] Yu SJ Gao W Shi W Yuan L Shen AD Yao KH [Nasopharyngeal carriage rate, antimicrobial resistance and serotype distribution of Streptococcus pneumoniae among children with upper respiratory infection]. Zhongguo Dang Dai Er Ke Za Zhi. 2014;16:988–92. Chinese. 25344177

[50] Tian J Li L Chen L Lu B [Distribution characteristics and drug sensitivity analysis of haemophilus influenzae in children with lower respiratory tract infection in Jiaxing area from 2015 to 2019]. Clinical Education of General Practice. 2020;17:431–4. Chinese.

[51] Mu L Li J Kuang L Zhou W Su M Dai W [Distribution characteristics and drug resistance analysis of Staphylococcus aureus infection in children from 2011 to 2017]. Chongqing Yixue. 2019;48:1241–4. Chinese.

[52] Leshem E Maayan-Metzger A Rahav G Dolitzki M Kuint J Roytman Y Transmission of Staphylococcus aureus from mothers to newborns. Pediatr Infect Dis J. 2012;31:360–3. 10.1097/INF.0b013e318244020e 22189535

[53] Chen Y Li L Wang C Zhang Y Zhou Y Necrotizing Pneumonia in Children: Early Recognition and Management. J Clin Med. 2023;12:2256. 10.3390/jcm12062256 36983257 PMC10051935

[54] Guo Y Yang D Cao Y Ding X Chen L Huo B Influence of COVID-19 public health restrictions on community-acquired pneumonia pathogens in children in Henan, China: a multicenter retrospective study. BMC Infect Dis. 2024;24:1381. 10.1186/s12879-024-10268-5 39627713 PMC11616279

[55] Lee CR Lee JH Park M Park KS Bae IK Kim YB Biology of Acinetobacter baumannii: Pathogenesis, Antibiotic Resistance Mechanisms, and Prospective Treatment Options. Front Cell Infect Microbiol. 2017;7:55. 10.3389/fcimb.2017.00055 28348979 PMC5346588

[56] Martin RM Bachman MA Colonization, Infection, and the Accessory Genome of Klebsiella pneumoniae. Front Cell Infect Microbiol. 2018;8:4. 10.3389/fcimb.2018.00004 29404282 PMC5786545

[57] Zinserling VA Swistunov VV Botvinkin AD Stepanenko LA Makarova AE Lobar (croupous) pneumonia: old and new data. Infection. 2022;50:235–42. 10.1007/s15010-021-01689-4 34472009 PMC8409273

[58] Xu A Zhu H Gao B Weng H Ding Z Li M Diagnosis of severe community-acquired pneumonia caused by Acinetobacter baumannii through next-generation sequencing: a case report. BMC Infect Dis. 2020;20:45. 10.1186/s12879-019-4733-5 31941459 PMC6964051

[59] Yazici Özkaya P Turanli EE Metin H Aydin Uysal A Cicek C Karapinar B Severe influenza virus infection in children admitted to the PICU: Comparison of influenza A and influenza B virus infection. J Med Virol. 2022;94:575–81. 10.1002/jmv.27400 34655235

[60] Shafik CF Mohareb EW Yassin AS Amin MA El Kholy A El-Karaksy H Viral etiologies of lower respiratory tract infections among Egyptian children under five years of age. BMC Infect Dis. 2012;12:350. 10.1186/1471-2334-12-350 23237512 PMC3538156

[61] Xie L Zhang B Xiao N Zhang F Zhao X Liu Q Epidemiology of human adenovirus infection in children hospitalized with lower respiratory tract infections in Hunan, China. J Med Virol. 2019;91:392–400. 10.1002/jmv.25333 30286268 PMC7159165

