# Supplementary material for: Clinical epidemiology of community-acquired pneumonia in children before, during, and after the COVID-19 pandemic, and independent risk factors analysis for severe community-acquired pneumonia
Source: J Glob Health. 2025 Sep 19;15:04212. doi: 10.7189/jogh.15.04212 (PMC12448372; doi:10.7189/jogh.15.04212)
Supplement: Online Supplementary Document [file jogh-15-04212-s001.pdf]

Supplement to: Li XO, Li LZ, An XJ, Tian J, Zhuoga B, Jin LH, Jin HJ, Li XF, Liu Y, Li JM, Mei WZ, Liu P, Pan JY, Lin ZT, Pang YS, Wu X, Peng Q, Hu XP, Su XW, Wang XN, Feng L, Zhang HT, Zhang DH, Yang ST, Lu ZP, Chen WQ, He B. Clinical epidemiology of community-acquired pneumonia in children before, during, and after the COVID-19 pandemic and independent risk factors analysis for severe community-acquired pneumonia. J Glob Health. 20xx;xx:xxx.

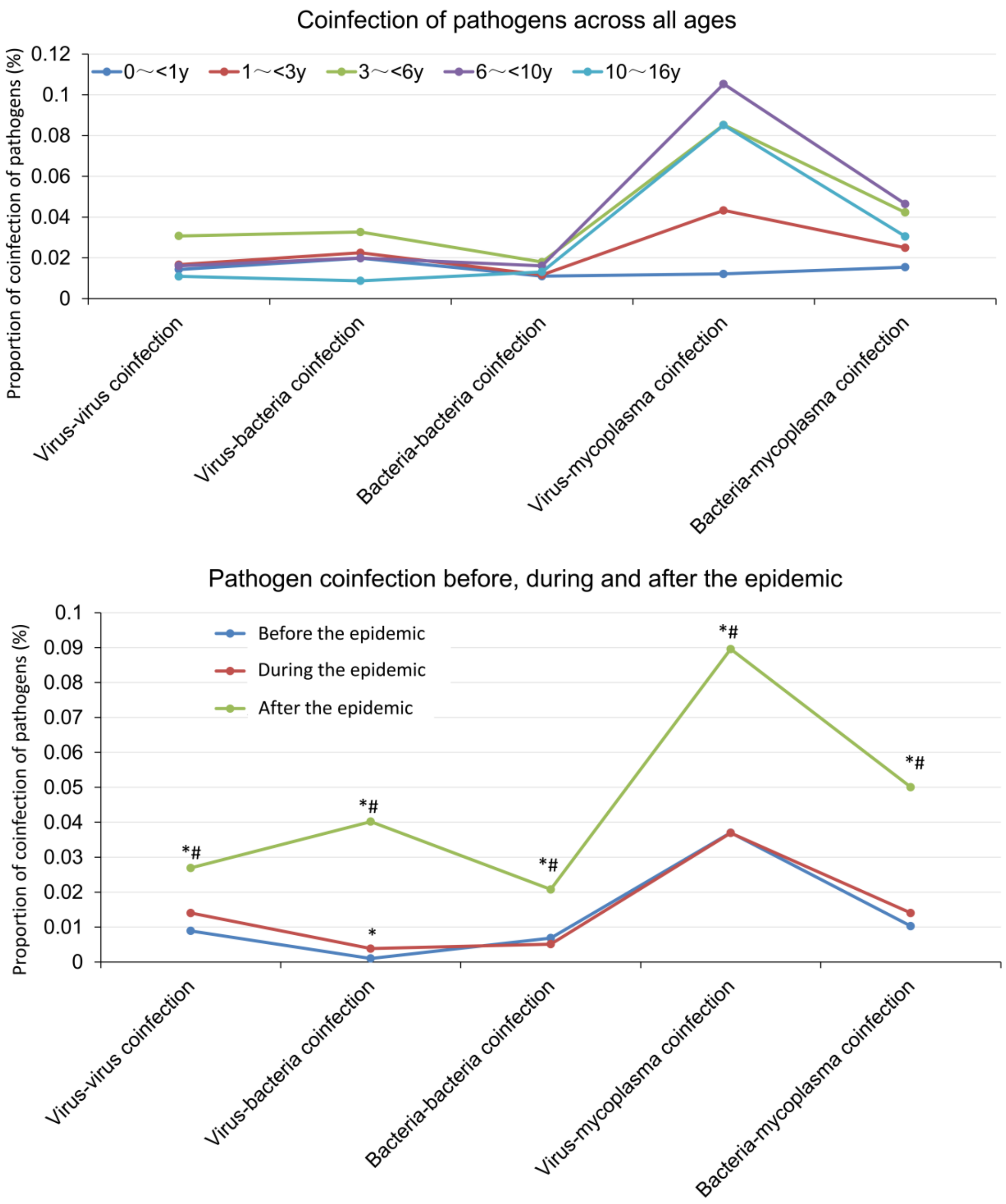

Figure S1. Coinfection of pathogens in different years and ages. *P < 0.05 VS before the epidemic; #P < 0.05 VS during the epidemic.

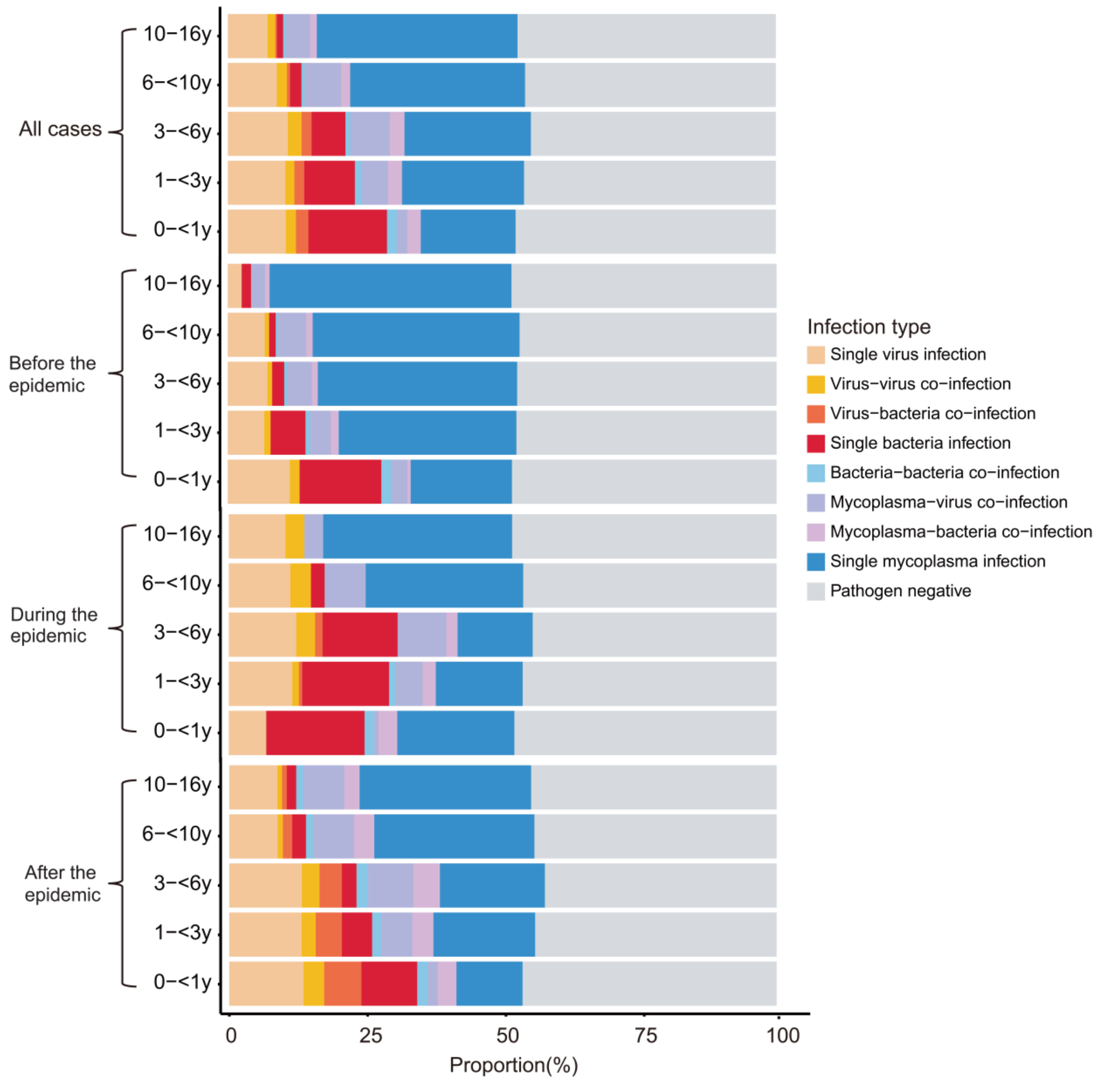

Figure S2. Pathogen coinfection of children with CAP in different years and of different ages. CAP – community-acquired pneumonia.

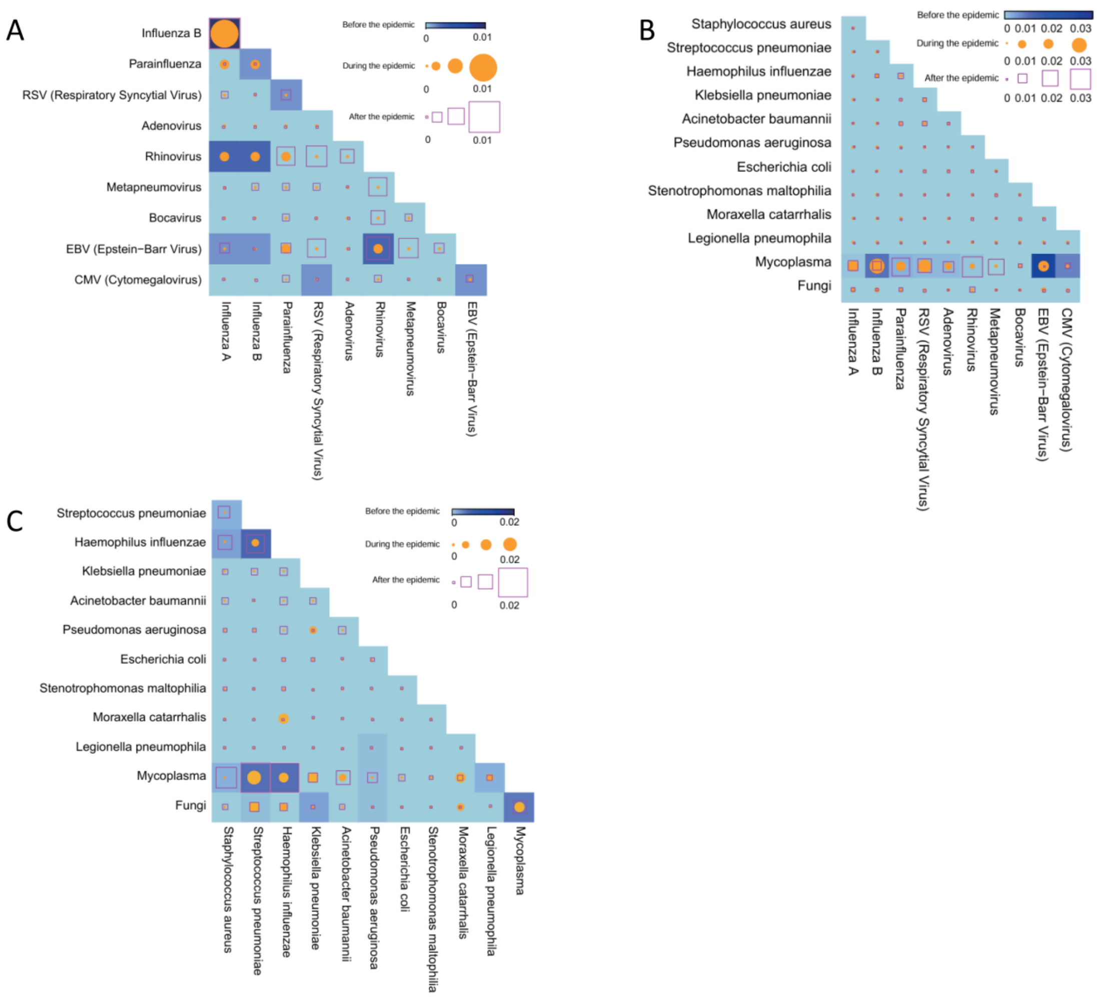

Figure S3. Heatmap of pathogen coinfection. **Panel A.** Heatmap of virus-virus co-infection. **Panel B.** Heatmap of virus-bacteria co-infection. **Panel C.** Heatmap of bacteria-bacteria co-infection.

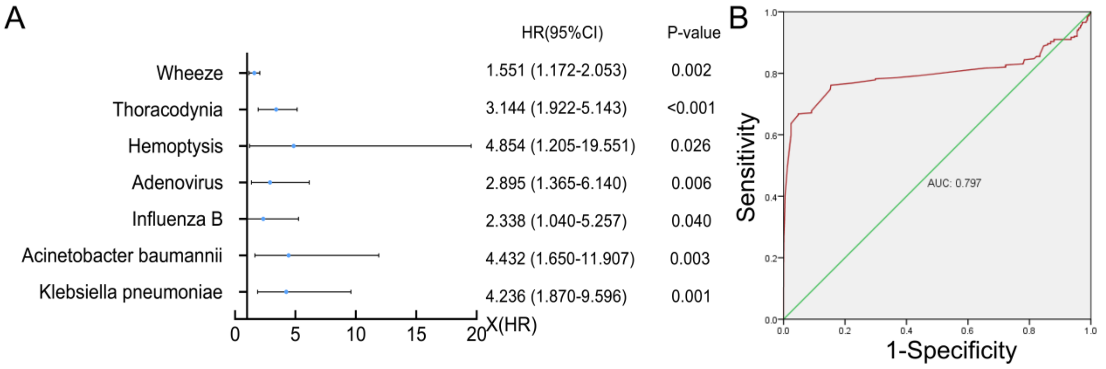

Figure S4. Risk factors and performance analysis. **Panel A.** Forest map of risk factors. **Panel B.** Receiver operating characteristic curve analysis.

Table S1. Analysis of pathogens in the study population

Pathogen	Before the pandemic (n = 1459)	During the pandemic (n = 785)	After the pandemic (n = 2936)	*P* value
Bacteria	72 (4.9)	76 (9.7)*	285 (9.7)*	<0.001
Virus	108 (7.4)	71 (9.0)	589 (20.1)†	<0.001
Mycoplasma pneumoniae	530 (36.3)	144 (18.3)*	1229 (41.9)†	<0.001
Fungus	23 (1.6)	14 (1.8)	48 (1.6)	0.934

Comparisons were conducted using Pearson's χ^2 test.
*$P < 0.05$, compared with before the pandemic.
†$P < 0.05$, compared with before and during the pandemic.
Data are presented as n (%).

Table S2. Comparison of clinical symptoms between CT score <8 and CT score ≥8 groups*

Characteristics	CT score <8 (n = 2440)	CT score ≥8 (n = 289)	*P* value
**Sex**			
Male	1310 (53.7)	153 (52.9)	0.810
Female	1130 (46.3)	136 (47.1)	
**Age, year, MD (IQR)**	4.8 (2.0–7.7)	4.3 (2.0–8.0)	0.840
**Chronic diseases**			
Allergic constitution	69 (2.8)	15 (5.2)	0.028
Recurrent respiratory infection	84 (3.4)	14 (4.8)	0.226
Heart disease	46 (1.9)	4 (1.4)	0.548
Cerebral dysplasia	11 (0.5)	0	0.619†
Epilepsy	25 (1.0)	5 (1.7)	0.240†
**Symptoms**			
Fever	1928 (79.0)	250 (86.5)	0.003
Cough	2388 (97.9)	274 (94.8)	0.001
Fatigue	197 (8.1)	32 (11.1)	0.082
Dyspnea	227 (9.3)	44 (15.2)	0.001
Wheezing	370 (15.2)	64 (22.1)	0.002
Thoracodynia	46 (1.9)	17 (5.9)	<0.001
Pharyngalgia	87 (3.6)	12 (4.2)	0.614
Hemoptysis	4 (0.2)	2 (0.7)	0.126†
Diarrhea	143 (5.9)	17 (5.9)	0.988
Celialgia	134 (5.5)	17 (5.9)	0.784

Vomiting	304 (12.5)	31 (10.7)	0.396
Rash	137 (5.6)	17 (5.9)	0.852
Convulsion	17 (0.7)	1 (0.3)	0.713†
**Complications**			
Respiratory failure	29 (1.2)	8 (2.8)	0.051†
Heart failure	20 (0.8)	4 (1.4)	0.312†
Myocardial injury	107 (4.4)	28 (9.7)	<0.001
Liver function injury	134 (5.5)	50 (17.3)	<0.001
Kidney function impairment	16 (0.7)	3 (1.0)	0.445†
Coagulation dysfunction	19 (0.8)	7 (2.4)	0.016†
Bronchiolitis obliterans	1 (0.04)	4 (1.4)	0.001†
Pneumothorax	2 (0.08)	0	1.000†
**Hospital stay, days, MD (IQR)**	8 (6–10)	10 (7–13)	<0.001
**Time from onset to pneumonia, days, MD (IQR)**	5 (4–8)	6 (4-8)	0.725
**Peak body temperature, °C, MD (IQR)**	39.0 (38.5–39.5)	39.0 (38.5–39.4)	0.063
**Total fever duration, days, MD (IQR)**	5 (3–7)	5 (4–7)	0.008
**Lung consolidation**	635 (26.0)	151 (52.2)	<0.001

IQR – interquartile range, MD – median

*Data are presented as n (%) unless specified otherwise.

†Pearson's χ^2 test with Fisher's exact test. Other comparisons were conducted using Pearson's χ^2 test.

Table S3. Comparison of pathogens between CT score <8 and CT score ≥8 groups, n (%)

**Characteristics**	**CT score <8 (n = 2440)**	**CT score ≥8 (n = 289)**	***P* value**
**Bacteria**	198 (8.1)	21 (7.3)	0.616
Staphylococcus aureus	32 (1.3)	2 (0.7)	0.574*
Streptococcus pneumoniae	88 (3.6)	6 0(2.1)	0.177
Haemophilus influenzae	78 (3.2)	3 (1.0)	0.041
Klebsiella pneumoniae	8 (0.3)	6 (2.1)	0.002*
Acinetobacter baumannii	7 (0.3)	4 (1.4)	0.022*
Pseudomonas aeruginosa	11 (0.5)	0	0.619*
Escherichia coli	3 (0.1)	0	1.000*
Stenotrophomonas maltophilia	2 (0.1)	0	1.000*
Moraxella catarrhalis	12 (0.5)	0	0.628*
Legionella pneumophila	2 (0.1)	1(0.3)	0.285*
**Virus**	423 (17.3)	40(13.8)	0.134

Influenza A	16 (0.7)	4(1.4)	0.154*
Influenza B	21 (0.9)	6(2.1)	0.059*
Parainfluenza	76 (3.1)	2(0.7)	0.019
RSV	31 (1.3)	4(1.4)	0.783*
Adenovirus	19 (0.8)	7(2.4)	0.016*
Rhinovirus	84 (3.4)	4(1.4)	0.061
Metapneumovirus	41 (1.7)	1(0.3)	0.122*
Bocavirus	8 (0.3)	0	1.000*
Epstein-Barr virus	139 (5.7)	19(6.6)	0.546
Cytomegalovirus	28 (1.1)	2(0.7)	0.764*
Mycoplasma pneumoniae	1180 (48.4)	152(52.6)	0.173
**Fungus**	33 (1.4)	5(1.7)	0.592*

*Pearson's χ^2 test with Fisher's exact test. Other comparisons were conducted using Pearson's χ^2 test.
